# A Hybrid VLC-RF Portable Phasor Measurement Unit for Deep Tunnels

**DOI:** 10.3390/s20030790

**Published:** 2020-01-31

**Authors:** Ismael Soto, Rafael Nilson Rodrigues, Gabriel Massuyama, Fabian Seguel, Pablo Palacios Játiva, Cesar A. Azurdia-Meza, Nicolas Krommenacker

**Affiliations:** 1 Department of Electrical Engineering, Universidad de Santiago de Chile, Santiago 9170124, Chile; fabian.seguelg@usach.cl; 2 Academic Department of Electrotechnic, Federal Institute of Santa Catarina, Florianópolis 88020-300, Brazil; rafael@ifsc.edu.br (R.N.R.); gabriel.am@aluno.ifsc.edu.br (G.M.); 3 Department of Electrical Engineering, Universidad de Chile, Santiago 8370451, Chile; pablo.palacios@ug.uchile.cl (P.P.J.); cazurdia@ing.uchile.cl (C.A.A.-M.); 4 Université de Lorraine, CRAN, CNRS UMR 7039, BP 70239 Nancy, France; nicolas.krommenacker@univ-lorraine.fr

**Keywords:** deep tunnels, global positioning system, location, mining sites, phasor measurement units, synchrophasors, visible light communication

## Abstract

In this manuscript we propose a hybrid Visible Light Communication and Radio Frequency (VLC-RF) scheme for the implementation of a portable Phaser Measurement Unit (PMU) for deep underground tunnels. Through computer simulations and laboratory measurements we are capable of providing Coordinated Universal Time (UTC) to the PMUs, as well as high accuracy positioning in a Global Positioning System (GPS) denied environment. The estimated PMU position, time stamp, and electrical power system measurements are sent to a central monitoring station using a radio frequency uplink with a data rate of hundreds of Kbps. Simulations and experimental measurements show that the proposed scheme can be used to control a large number of VLC-RF PMU devices inside a tunnel. The tests demonstrate the viability of the hybrid prototype, which will improve performance compared to commercial PMUs that lack these features.

## 1. Introduction

A Phaser Measurement Unit (PMU) makes part of a Synchronized Phasor Measurement System (SPMS). A PMU is a device used to estimate the magnitude and phase angle of an electrical phasor quantity, as well as for oscillation monitoring, for the purpose of improving the observability of the Electric Power System (EPS) [1]. It has a high data rate acquisition and uses a GPS in order to synchronize its measurements. The SPMS allows the dynamic performance assessment of an EPS [2,3,4], enabling the detection of disconnected transmission lines or islanded subsystems, supervision of voltage stability, and assisting system analysis and restoration in the event of more severe disturbances [1]. Many systems based on the Supervisory Control and Data Acquisition (SCADA) scheme are used for monitoring dynamic conditions of the EPS, but are not capable of detecting fast events due to their low data acquisition rate. Therefore, a SPMS allows monitoring the dynamic performance under normal operating regimes, as well as during periods of more severe disturbances, making possible to visualize very fast transient effects that may not be detected using a conventional monitoring structure [5]. The SPMS also uses a Phasor Data Concentrator (PDC) to store data, which allows monitoring and analysis of the EPS.

Despite the high cost of PMUs, their usage in distribution systems is becoming more frequent [6]. The rising use of PMUs and their high cost justify the development of low cost PMUs for EPS monitoring.

Electrical power systems in underground mines have many substations, which makes them very similar to a distribution system. Therefore, the use of PMUs in this type of environment is a reliable choice. Currently, detailed electrical measurements in mines are limited only to equipment and structures located on surface.

Operational conditions of the EPS depend on the dynamic behaviour of electric machines, oscillations, and disturbances. The previously indicated characteristics may compromise its proper functioning. In this scenario, PMUs are used for monitoring large mining machines located in deep tunnels and throughout the mine, enabling power quality analysis and fault detection. This would not only allow to take actions in order to improve the machines efficiency and performance, but also could be a way of detecting non-operating machines, reducing economic losses and helping to locate possible critical issues such as floods or landslides.

However, a major concern emerges in underground scenarios. Inside tunnels, GPS signal used by the PMU to synchronize the acquired data is not available. Traditional GPS systems do not work in indoor scenarios because satellites’ signals cannot penetrate to confined places [7]. To overcome this limitation, some other alternatives have been proposed for localization in underground environments, such as Zigbee, Wi-Fi, Bluetooth, among others [8]. The previous technologies normally use a portion of the Radio Frequency (RF) electromagnetic spectrum to provide wireless communication. RF signals face some constraints when used in underground mines. RF signals are affected by a large amount of reflections, scattering, and shadowing. This severely affects propagation performance of these technologies [8].

Visible light communications (VLC) [9] is a novel technology based on Optical Wireless Communication (OWC) systems that can be used hybridized with RF without causing interference [10,11]. VLC uses Light-Emitting Diodes (LED) to transmit data at high speeds, while Photo-Detectors (PDs) are used at the receiver side to detect the variation on the optical power [12,13]. This method is known as Intensity Modulation and Direct Detection (IM/DD). Recently, high efficiency and brightness LED devices have been developed. Moreover, bandwidth of hundreds of Mbps [14] have been reached. Nowadays, several applications of this novel technology are being studied, such as Light Fidelity (Li-Fi) [15], satellites’ communications, and vehicular communications, among others [15]. Among them, Visible Light Positioning (VLP) has gained the attention of researchers due to its merits in terms of accuracy, cost, safety, and reliability for indoor environments [16,17].

Recently, we have addressed the problem of positioning in underground mines using VLC [18,19,20,21]. In this article, and to the authors’ best knowledge, we present for the first time the usage of VLC to provide time and position information in GPS format to distributed PMUs inside an underground tunnel.

The document is organized as follows: in Section 2, the discussion is focused on positioning and communications technologies for tunnel environments. In Section 3, the proposed system description is provided. Section 4 details the materials and methods used for positioning and time synchronization of the PMU devices. In Section 5, the experimental results at laboratory scale are presented. Finally, main conclusions of this study are delivered in Section 6.

## 2. Technologies Used to Provide Positioning and Wireless Communication in a Tunnel

Numerous technologies can be used to provide positioning in a tunnel. Each technology has its own merits in terms of performance, coverage, costs, among others. Hence, the idea of combining different types of location technologies to overcome difficulties and/or improve system performance arises. The main problem with hybrid systems is that there is no unified systematic evaluation of the possible advantages and disadvantages of using different technologies simultaneously for location purposes. Next we will discuss technologies based on other types of signals.

- Acoustic systems: Acoustic signals are mechanical waves. The estimation of the acoustic position can be based on ultrasound or audible sound. The estimated range of acoustic positioning systems is about 10 m due to the specific decay profile of the channel. Trilateration-based methods can be easily implemented using these types of systems using a microphone array on the receiver side. Acoustic systems are severely affected by multipath propagation, directivity of transducers and noise. In addition, increasing the signal strength is not a viable alternative, since a huge amount of energy is required, reducing the battery life [22].
- Inertial Navigation Systems (INS): INS estimates the position, speed and orientation of the mobile node from an Inertial Measurement Unit (IMU). This type of systems operates without infrastructure requirement. When the initial position and orientation of the mobile device are known, the subsequent positions, orientations and speeds can be continuously updated by the Dead Reckoning (DR) without the need for external reference positions. The accuracy of the position propagated depends largely on the quality of the position and orientation that was initially provided. Accelerometer measurements are also affected by noise. Because of this, the INS decreases its accuracy over the distance traveled, this being its main problem [23].
- Signage and maps: Signage and maps are traditionally the most available approach for human navigation in indoor environments. The actual position is provided by static signaling and, to help the mobile user reach his destination. Today, new signs and maps are replacing this traditional approach. For example, cameras and mobile devices can replace the user’s vision and reasoning to provide positioning and navigation. The use of a camera to scan unique “markers” is proposed. By doing so, the single mark provides information to a mobile phone application to determine the actual position [24].
- Magnetic field: Unlike electromagnetic waves, magnetic waves are able to penetrate the interior walls of buildings. Because of this, magnetic field based location systems do not suffer from several disadvantages found in electromagnetic systems. The second type of location systems based on magnetic fields use artificially generated magnetic fields. In this type of systems a variable electric current is used that circulates in concentric coils to create the artificial and controlled magnetic field (Lenz effect). Usually, artificially induced magnetic fields have a short coverage range (less than 3 m) and, due to this, their application is limited to small volumes. This means that there is no feasibility of using these systems for underground mining [25].

The focus of this discussion is on VLC technologies used for localization inside a tunnel. Other wireless technologies, such as Bluetooth [26], Wi-Fi [27], and GPS/PMU location schemes that are standardized [28], are not discussed in this section.

VLC systems are commonly used for downlink since visible light causes discomfort to humans who are close to the light source when used for uplink [29]. In general, range free algorithms can be classified into two classes: single or multi hop algorithms. The first category assumes infrastructure network topology (one to many), where single hop two-way communication between transmitter and receiver is done. In the second category, ad-hoc networks (many-to-many) with multiple hops between the anchor and mobile nodes are assumed. Since pure VLC devices are not capable to provide two-ways data transmission, one hop methods have been mainly used in VLP systems. As mentioned above, uplink is done by means of RF communication.

Among all wireless communication technologies used along with VLC, certain RF and Infrared (IR) technologies are usually used to support uplink communication. IR communications have a short range of coverage (about 10 m). On the other hand, certain RF technologies have shown to cover a large area inside tunnels. Different RF bands have been studied inside tunnels. Further details regarding channel modelling and signal propagation characteristics of RF in underground mines can be found in [30].

In Emslie et al., the propagation of RF signals between 500–1000 MHz was studied. The experimental study showed that attenuation of signals in this frequencies is relatively low in straight mine entries [31]. On the other hand, it has been demonstrated by real measurements that the Medium Frequency (MF) band (300 kHz–3 MHz) has a larger coverage and less attenuation compared to the Ultra High Frequency (UHF) band and higher frequency bands [30].

Higher frequencies, such as UHF, Very High Frequency (VHF), and Super High Frequency (SHF) propagate in Line Of Sight (LOS) and 300 m down a mine entry. Considering state-of-the art literature on this type of systems, in terms of theoretical and experimental results, UHF systems may offer a larger coverage area in straight and unobstructed tunnels [30]. Moreover, UHF based technologies are an interesting proposal for the mining industry due to their low cost, small form factor, scalability, and availability in off-the-shelf devices. In addition, their coverage and other propagation aspects (e.g., requiring LOS between Tx and Rx) can be resolved by appropriate antenna and wireless network designs. Therefore, taking into consideration propagation aspects of RF signals inside mine tunnels, in this proposal we choose a technology capable of providing communication in the UHF band (300 MHz to 3 GHz).

## 3. System Description

**Figure 1:**
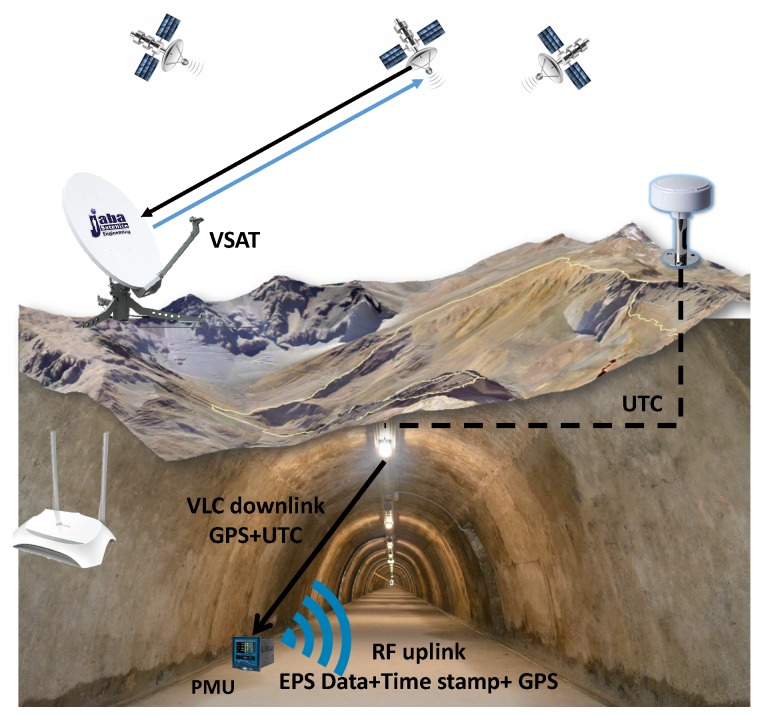
Integration diagram of Phasor Measurement Unit (PMU) and Visible Light Communication and Radio Frequency (VLC-RF) system in a mine.

**Figure 2:**
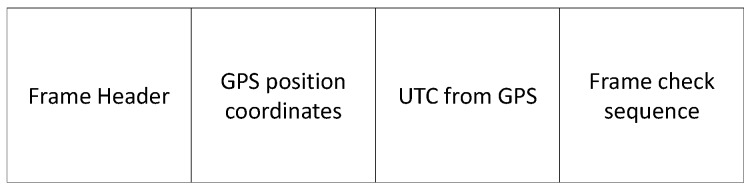
Transmitted information by VLC.

In Figure 1, a general description of the proposed system is illustrated. The Global Navigation Satellite System (GNSS) provides the UTC to coordinate the samples acquired from different PMUs. This first stage is done by a low cost GPS device from which the time stamp is retrieved. Time and position information travels throughout the backbone network. Then, VLC cells are used to deliver the information wirelessly to the PMU. The packet to be broadcasted by the VLC cell is depicted in Figure 2. We use VLC as the downlink technology due to its capability to provide high accuracy positioning and its low cost implementation since it uses the already deployed lighting infrastructure to provide data transmission and illumination, as detailed in Section 2. Once the PMU takes a sample from the EPS, data containing universal time stamp, electrical power system measurements, and PMU position information is delivered to a central monitoring station by using a long range RF link. As it has been demonstrated in previous studies, UHF RF links can achieve a large coverage in underground tunnels, and the transmission of the signal can be considered as a wave-guide [8]. Therefore, the infrastructure required to retrieve the data from the PMUs is reduced. A Very Small Aperture Terminal (VSAT) link is used in case of an earthquake [32], due to the collapse of the communication infrastructure in emergency cases [33].

## 4. Materials and Methods

Figure 1 shows the diagram of the proposed scheme that combines PMUs and a VLC-RF system in a mine. The PMU’s analog inputs are processed by a set of anti-aliasing filters to condition the signal and restrict the signal bandwidth to satisfy part or all of the Nyquist-Shannon sampling theorem at a bandwidth of interest, before being sent to the Analogic-Digital Converter (ADC). The GPS-like position and UTC time stamp received from the VLC cell are used as input to the processing unit, which assembles the protocol containing the approximated position in latitude and longitude coordinates, time stamp, and other control symbols.

### 4.1. Phasor Measurement Unit

**Figure 3:**
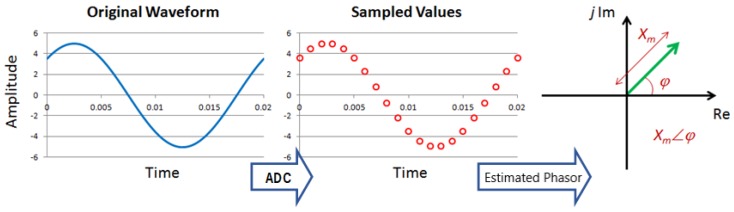
Phasor estimation process of a PMU.

Specifically, a PMU digitizes a voltage and/or current signal, and estimates the phasors (amplitude and phase angle) synchronously to a UTC time base. The phasors obtained in this timing are called synchrophasors. Figure 3 illustrates this process.

A typical PMU normally executes three actions: data acquisition, processing, and time synchronization. The ADC requires data acquisition of at least 15360 samples per seconds for a 60 Hz electrical system [4]. The sampling of the voltage for each of the three phases is done simultaneously. The processing unit estimates the positive sequence value of all voltage signals, including the suitability of data transmission information in the format specified in standards C37.118.1 [34] and C37.118.2 [35].

The timing system provides the time reference for the acquisition and suitability of the measured signal. This reference is provided by the GPS. Since deep tunnels are GPS denied environments, we distribute this time reference through the tunnel by means of a VLC network. After the data is acquired and processed with its time information, it is sent to a PDC via a long range wireless communication. The PMU prototype in this work is based on the STM32F429 Nucleo-144 board, as specified in [4].

### 4.2. Visible Light Communication System

Channel modeling is an important step for an efficient, reliable, and robust VLC system design. Many studies have been conducted to characterize wireless optical channels, but there is no generic channel model for VLC systems in underground mining. The recursive method was first proposed by Barry et al. [36] in the infrared spectrum. Subsequently, Barry’s model was extended to the visible light spectrum [37].

**Figure 4:**
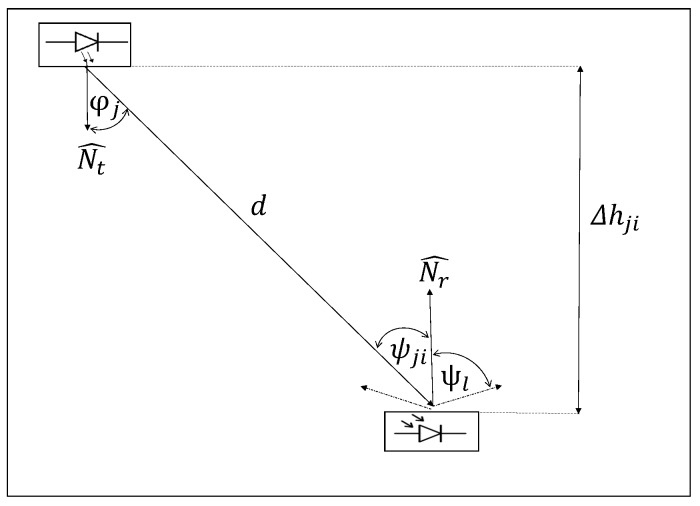
VLC channel model.

Figure 4 represents the signal received by the PMU, modelled as a simple VLC channel, using several filters in the receiver, so as to detect only one frequency. The signal received by the PMU is given as follows

(1) $$P_{i}=R_{PD}\cdot P_{j}\cdot H_{ji},$$

where $P_{i}$ is the power received by the PMU *i*, wherea $R_{PD}$, $P_{j}$, and $H_{ji}$ are the PD response, the power transmitted by LED *j*, and the channel gain of LED *j* to the mobile node *i*, respectively. In the case of an LOS link, the channel gain $H_{ji}$ can be calculated as [37]

(2) $$H_{ji}=\left\{\begin{array}{cc} \frac{(m_{l}+1)A}{2\pi d^{2}}\cos^{m_{l}}\left(\varphi_{j}\right)G\left(\psi_{ji}\right)\cos\left(\psi_{ji}\right) & 0\leq \psi_{ji}\leq \Psi_{l}\\ 0 & \mathrm{elsewhere} \end{array}\right.,$$

where $m_{l}$ is the Lambertian order transmission of the LED light, *A* is the effective area of the PD. Whereas *d*, $\varphi_{j}$, and $\psi_{ji}$, are the distance, angle of irradiance, and the angle of incidence of the signal, whilst $G\left(\psi_{ji}\right)=T_{s}\left(\psi_{ji}\right)\cdot g\left(\psi_{ji}\right)$ is the gain of the optical filter and gain of the optical concentrator between transmitter *j* and receiver *i*, respectively. Finally, $\Psi_{l}$ is the Field of View (FOV).

The above is related to the height between the transmitter, the plane of the receiver, and the FOV of the receiver. It is also assumed that can be adjusted with the FOV of the receiver. Thus, satisfying the overlay condition. The coverage radius of each VLC cell $R_{j}$ is determined by $R_{j}=tan\left(\Psi_{l}\right)\Delta h_{ji}$, where $\Delta h_{ji}$ is the height between the transmitter and receiver.

**Figure 5:**
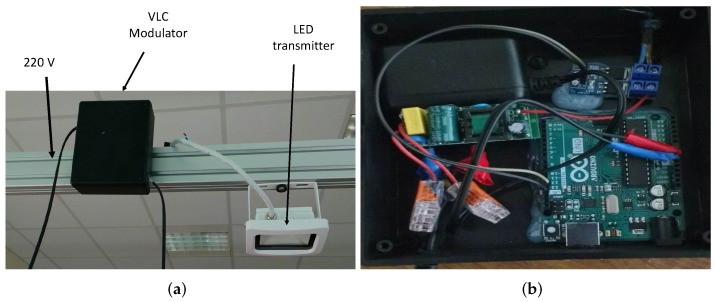
VLC transmitter (**a**) general overview and (**b**) modulator in detail.

In order to provide position and time information, a low cost VLC device is implemented. The proposed device uses on-off-keying modulation to broadcast necessary information. In Figure 5, the proposed VLC device is depicted.

**Figure 6:**
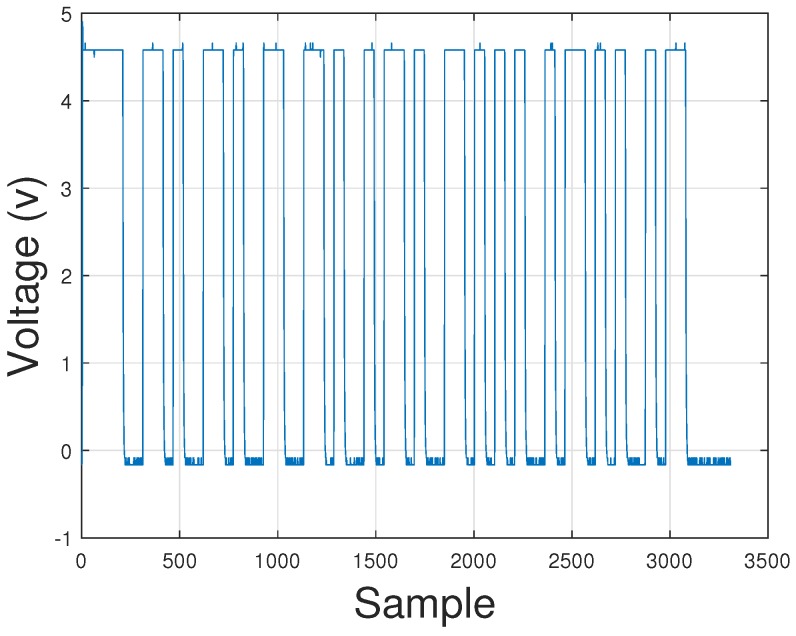
Digitalized VLC packet at PMU side.

At the receiver side, the optical modulated signal is converted to a voltage signal by using a photodetector and a trans-impedance amplifier. In this proposal we use the Vishay BPW21R photodiode and the OPA657 amplifier [38]. The voltage is passed through an ADC converter in order to demodulate the VLC packet. In Figure 6, the digitized signal received by the photodetector is shown.

**Figure 7:**
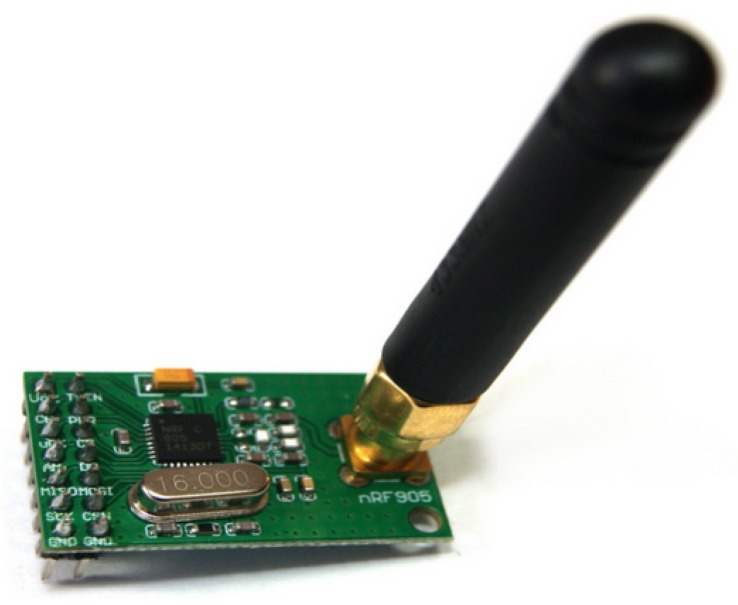
Single chip Ultra High Frequency (UHF) transceiver nRF905.

Data demodulated is passed directly to the PMU. Different low cost devices are capable to provide wireless communications using the UHF frequency band. In this work, we chose the low cost wireless transceiver nRF905 device. This transceiver is a single chip communication device that can work in different UHF bands: 433, 868, and 915 MHz. It has an adjustable output power up to 10 dBm, and low current consumption on transmit (9 mA) and receive mode (12 mA). In Figure 7 the transceiver nRF905 is shown. More details regarding the transceiver can be found in [39].

### 4.3. Single Hop Range Free Methods

In general, range free methods provide low cost, low complexity, and large scale position estimation. However, they suffer from low accuracy. In underground tunnels the accuracy of the method is not a severe problem and, localization errors up to 60 m are accepted for personnel and mobile devices [40]. In this section the range free VLC positioning method used for PMU positioning is explained in detail.

#### 4.3.1. Cell of Origin

Cell of Origin (COO), also known as cell ID, is a range free positioning method. COO estimates the location of the PMU by using the geographic coordinates associated with the serving cell. Therefore, the position of the PMU is estimated as the centroid of the VLC cell coverage area as follows

(3) $$\left({\hat{x}}_{PMU},{\hat{y}}_{PMU}\right)=\left(x_{j},y_{j}\right),$$

where $x_{j}$ and $y_{j}$ are the position of the VLC cell to which the PMU is connected. This information is received in the VLC packet given in Figure 2.

#### 4.3.2. Convex Position Estimation

Another approach is to locate the mobile nodes using the connectivity information in order to estimate a convex region where the mobile device could be located. Convex Position Estimation (CPE), proposed by Doherty et al., uses connectivity information in order to find the minimum bounding rectangle of a connection-based set. CPE reduces the size of the feasible connectivity set using the spatial reuse characteristic of PDs [18,41]. This minimum rectangle, also called bounding box, is found by solving iteratively a linear optimization problem with connectivity constraints. The bounding box is a convex set in $R^{2}$, since the rectangle is the intersection of four halfspaces, hence is a convex set in $R^{2}$ [42]. The CPE optimization problem is stated as follows

(4) $$\left[2\right]\in R^{2}D^{T}\hat{x},∥\hat{x}-C_{j}∥\leq R_{j},$$

where $D\in R^{2}$ is the vector which takes the values D=[10],D=[−10],D=[01],andD=[0−1] in order to find the minimum and maximum of the bounding box, while $C_{j}=\left(x_{j},y_{j}\right)$ is the vector that contains the jth LED position.

The restriction of the CPE positioning problem given in Equation (4) states that a feasible estimated localization point $\hat{x}=\left(x_{i},y_{i}\right)$ must lie inside the connectivity area. Finally, the estimated position of the PMU is computed as follows

(5) $$\left({\hat{x}}_{PMU},{\hat{y}}_{PMU}\right)=\left(\frac{{\hat{x}}_{min}+{\hat{x}}_{max}}{2},\frac{{\hat{y}}_{min}+{\hat{y}}_{max}}{2}\right),$$

where ${\hat{x}}_{min}$, ${\hat{x}}_{min}$, ${\hat{y}}_{min}$. and ${\hat{y}}_{max}$ are the bounding points of the rectangle.

#### 4.3.3. Centroid

The Centroid scheme was proposed by Bulusu et al. in [43]. This localization scheme assumes that a set of *M* connected LED anchor nodes, with overlapping regions of coverage, exist in the deployment area of the VLC network. The main idea is to treat the VLC nodes, located at $\left(x_{j},y_{j}\right)$, as point masses. In the most general form, the “mass” of each anchor node is considered to be equal, and the estimated mobile node’s position is obtained as follows

(6) $$\left({\hat{x}}_{PMU},{\hat{y}}_{PMU}\right)=\left(\frac{1}{M}\sum_{1}^{M}x_{j},\frac{1}{M}\sum_{1}^{M}y_{j}\right)$$

This method does not requires any ranging estimation and depends fully on network connectivity. Moreover, coordination between the unknown node and anchor node is not required. Therefore, implementation of the centroid algorithm is relatively simple.

## 5. Results and Discussion

In this section, experimental results are presented to validate the correct functioning of the PMU in Brazil and Chile, as well as the methods described in Section 4.

### 5.1. Analysis and Results of the PMU

**Figure 8:**
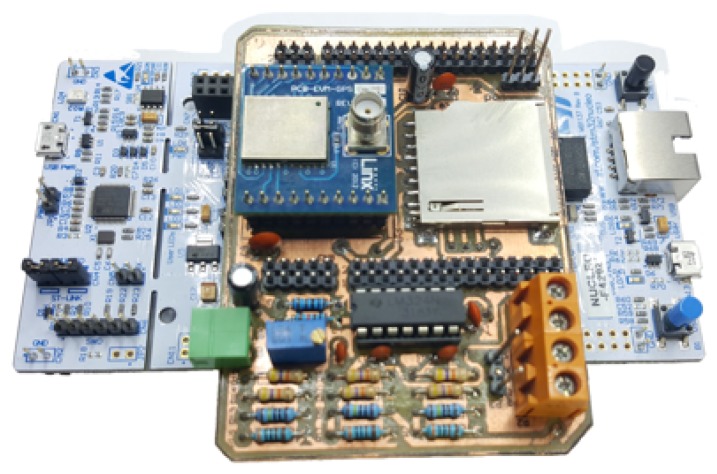
PMU prototype developed at labsmart/Federal Institute of Santa Catarina (IFSC).

The results are based on prototypes developed in labsmart/IFSC using STM32F429, the GPS module RXM-GNSS-GM LOT GR1749 [44], and a voltage divider, as shown in Figure 8. More details can be found in previous works [4]. The measurements presented were made at the campus of IFSC in Florianópolis (Brazil), UTEM (Universidad Tecnológica Metropolitana) in Santiago (Chile), and at USACH (Universidad de Santiago de Chile). The measurements taken at USACH correspond to its campuses located in the cities of Iquique and Santiago. The results from Brazil are verified to check that the PMU prototype is working well at 60 Hz. Whereas the PMU prototype installed at USACH (Santiago campus) is then compared to the PMUs installed at UTEM and USACH (Iquique campus) at 50 Hz.

**Figure 9:**
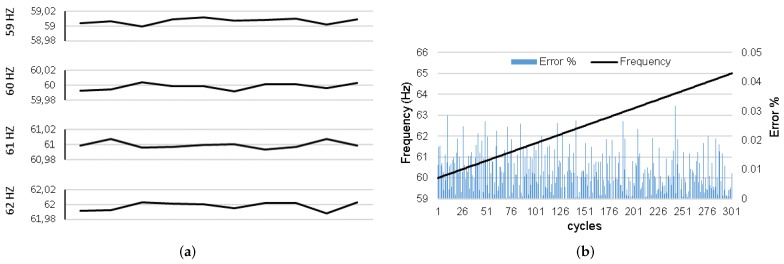
(**a**) Estimate for different frequency levels. (**b**) Frequency estimation error for 1 Hz/s ramp input.

Figure 9a,b highlights some important aspects. The readings are based on a single phase 3 Vpp signal and 1.5 Volts offset using the Tektronix AFG1022 function generator with ±(1+1mVpp) accuracy. Figure 9a illustrates the frequency estimate values in 10 cycles from 59 Hz to 62 Hz. In all cases the errors are less than 0.02 Hz. Figure 9b shows the frequency values for a frequency ramp from 60 Hz to 65 Hz at a rise rate of 1 Hz/s. The largest observed error is 0.032% at 64.033 Hz. For the other frequencies, the errors are less than 0.030%.

**Figure 10:**
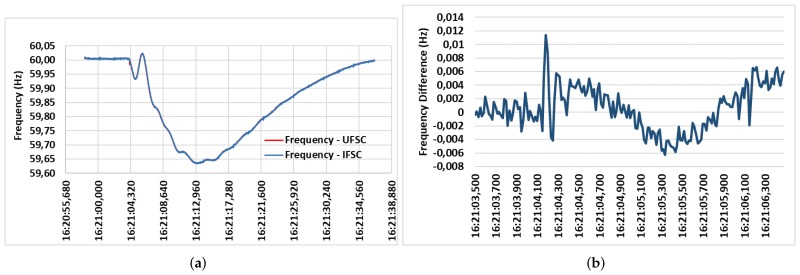
(**a**) Frequency variation during disturbance. (**b**) Measurement differences between the PMU at IFSC and PMU at University of Santa Catarina (UFSC).

Figure 10 illustrate comparisons of the PMU prototype installed at IFSC to a commercial Reason DR60 Digital Fault Recording PMU installed at the University of Santa Catarina (UFSC), both in Florianópolis, Brazil. Geographically, the institutions are very close, therefore; no frequency differences in the network between the equipment can be considered.

Figure 10a shows some results of frequency fluctuations due to a disturbance occurred on 17 February 2018. Frequency values range from 60.02 Hz to 59.63 Hz. Measurements between PMUs are similar, showing satisfactory functioning of the proposed prototype. Figure 10b illustrates the differences in frequency values measured by the PMU IFSC and the PMU UFSC. The highest absolute frequency error is 0.01135 Hz, occurring at 16:21:04.183 UTC. The measurements are very similar between PMUs.

**Figure 11:**
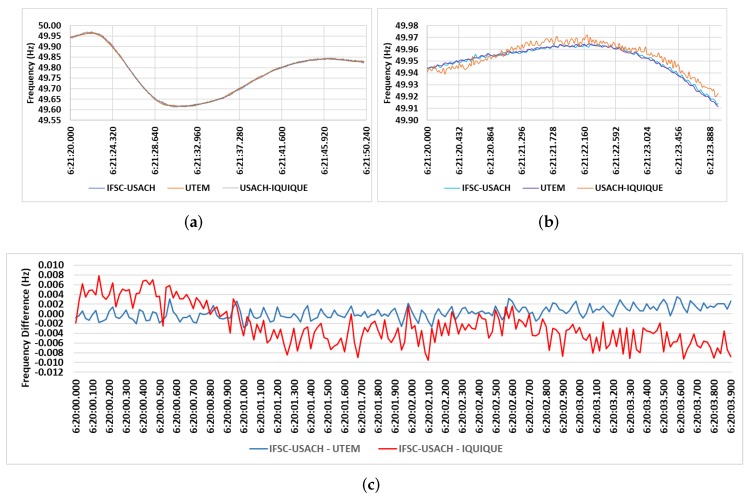
(**a**) Frequency measurements of the PMU IFSC-USACH, PMU UTEM, and PMU Iquique. (**b**) Highlight of frequency values measured by the PMU IFSC-USACH, PMU UTEM, and PMU Iquique. (**c**) Frequency difference between PMU IFSC-USACH and PMU UTEM as well as between PMU IFSC-USACH and PMU Iquique.

Figure 11 depicts comparisons of the PMU prototype installed at USACH/Santiago with two commercial PMUs, specifically a SEL-351A1 PMU in UTEM and a GE RVP-311 PMU in USACH/Iquique. Finally, Figure 11a shows frequency values measured at USACH, Santiago/Chile, on 3 July 2019 between 06:21:20.000 and 06:21:50.240 UTC during a minor system disturbance. These values were also measured by the IFSC PMU prototype, by the UTEM PMU and by the USACH/Iquique PMU. In particular, Figure 11b highlights frequency values measured between 06:21:20.000 and 06:21:24.000 UTC, whereas Figure 11c shows the frequency difference values between the IFSC PMU prototype and the commercial PMUs installed at UTEM and USACH/Iquique.

From Figure 11 it’s possible to observe that the measured frequency values are very similar. The largest frequency difference in modulus of the values measured between the PMU IFSC-USACH and the other commercial PMUs were 0.00522 Hz at 6:20:04.500 for the PMU UTEM and 0.00965 Hz at 6:20:16.400 for the PMU Iquique. The largest values differences for the PMU at Iquique possibly occurred due to small electromechanical oscillations between generators, as Santiago and Iquique are 2000 km apart.

In relation to the VSAT link in Figure 1, it is used to send the protocol described in Figure 2 to the command center located on the surface or inside the tunnel and the central offices that are in Santiago. For that reason, measurements were made between the PMUs in Santiago and Iquique, which is the area where most of the underground copper and lithium mines are located. Obviously, the protocol in Figure 2 needs more headers and parity checks to protect the information when the PMUs are mobile, but in this industrial application it is not necessary according to the disccusion given in Section 3. The excessive protection of the communication protocol in the end restricts the number of PMUs that can be placed in the tunnel to around 750 [45]. If the number of PMUs increases above this level, it will be necessary to carry out studies with higher altitude and faster satellites.

### 5.2. Analysis and Results for Range Free Positioning Methods

**Figure 12:**
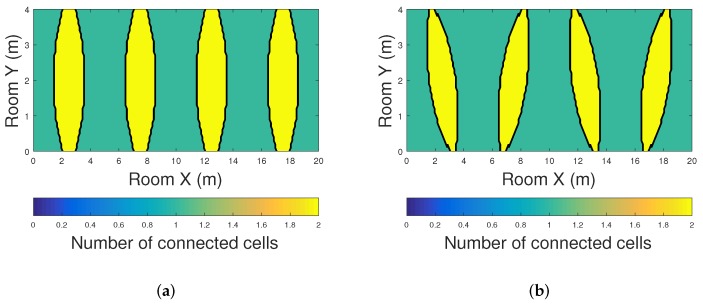
Coverage of VLC cells inside the tunnel using (**a**) linear LED arrangement and (**b**) zig-zag LED arrangement.

It is important to note that, due to the low lighting infrastructure deployed inside the tunnel, other positioning algorithms such as multilateration, trilateration or angulation cannot be used [29]. These methods require the detection of more than one beacon to estimate the position of the PMU, while the methods proposed in this article can localize the PMU by receiving VLC information from a single source. Whereas for range free methods, the main constraint these methods is that they largely depend on the network architecture (separation between the LEDs, arrangement configuration, LEDs density, among others). Due to this, two different lighting configurations that can be typically found in tunnels are evaluated in this manuscript: linear and zig-zag configurations. In Figure 12, the coverage of the VLC cells using linear and zig-zag arrangement is shown. As can be seen, for most of the proposed scenarios, the mobile node receives only one VLC beacon.

**Figure 13:**
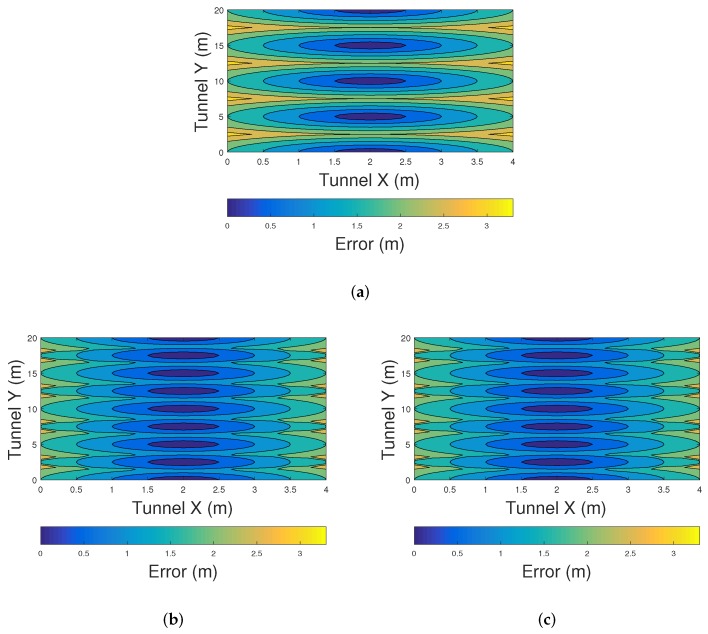
Error distribution in a linearly arranged VLC network using (**a**) COO, (**b**) CPE, and (**c**) Centroid.

Figure 13 shows the distribution of the positioning error using the COO, CPE, and Centroid methods in a linearly arranged VLC network. The scenario used for this experiment is similar to the tunnel shown in Figure 1. The LED lights are placed every of 5 m in the y-axis, while the tunnel section is 4 m wide and 20 m long. The results are taken from USACH’s laboratory-scale experimental measurements. Inter cell interference is prevented by interspersing two different frequencies assigned to the VLC sources, i.e., $VLC_{f_{1}}$ = 20 KHz, $VLC_{f_{3}}$ = 30 KHz, and $VLC_{f_{3}}$ = 20 KHz. Due to this, two different sources can be received at the same time.

Figure 13a shows the performance of the COO method. The error using this method decreases when the PMU is closer to a VLC source. On the other hand, large errors can be found when the PMU is located near to the walls of the tunnel. Figure 13b,c show the accuracy of CPE and centroid methods. The accuracy of these positioning algorithms is similar when there is a low density VLC network. In such scenarios, centroid methdos is preferred over CPE since its computational complexity is lower.

**Figure 14:**
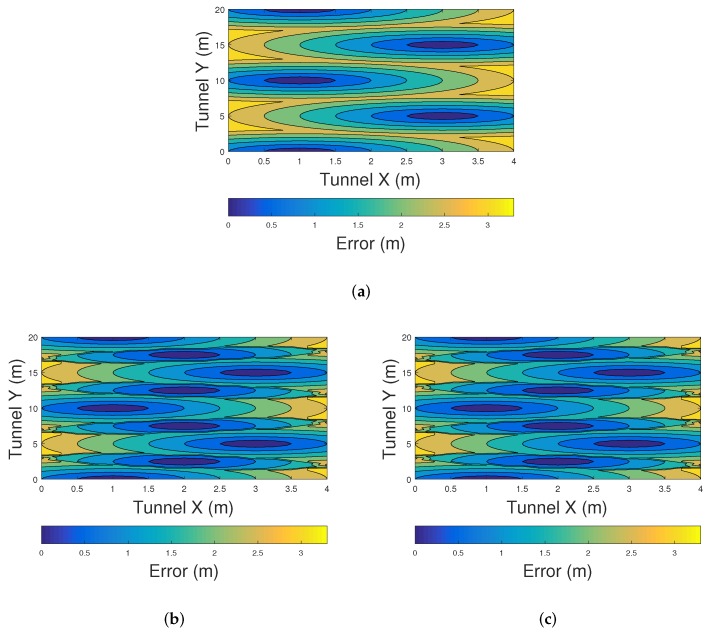
Error distribution in a zig-zag arranged VLC network using (**a**) COO, (**b**) CPE, and (**c**) Centroid.

Similarly, Figure 14 shows the error distribution of the three proposed methods using a zig-zag LED arrangement in a tunnel. Figure 13a shows the performance of the COO method. There exist a high detrimental effect on the position estimation when the sources are disposed in a zig-zag arrangement. Although in this scenario the number of LEDs is the same compared to the previous ones, the zig-zag disposition of LEDs increases the distance between beacons, and as a consequence, decreases the performance of the methods. Figure 14b,c show the errors distribution of CPE and Centroid methods. The impact of the network architecture in these methods in terms of accuracy is lower compared to COO method. Similarly to the results found for the linearly arranged VLC network, in the zig-zag scenario, CPE and centroid methods have the same accuracy.

Finally, we can conclude that when a low density network is used to provide range-free positioning, the centroid algorithm is preferred over COO and CPE since it achieves higher positioning accuracy with low computational complexity. Moreover, a higher position accuracy is achieved when using linearly arranged VLC cells.

## 6. Conclusions

In this work, a hybrid visible light/radio frequency (VLC-RF) communication scheme has been proposed for the implementation of a portable PMU for deep tunnels. Through computer simulations and laboratory measurements, we were able to deliver by means of VLC UTC information to the PMUs along the tunnel, as well as precise positioning in an environment where GPS signal is not available. Among the three evaluated positioning methods, the centroid algorithm outperforms CPE in terms of computational complexity and COO in terms of accuracy. This is for a scenario with low LED infrastructure density.Furthermore, a RF uplink transmits the PMU’s estimated position, time stamp, and power system measurements to a central monitoring station. The proposed scheme can be used to monitor a large number of portable PMU devices within a deep tunnel. Finally, technical feasibility of the hybrid prototype was proven. The proposed hybrid scheme is capable of enhancing performance when compared to a commercial PMU.

## 7. Patents

“Soto, I. and Lagos, C.”, Título: “Sistema y método de comunicación a través de luz visible para túneles subterráneos”. Country: CHILE, *N*°: CL3778-2015.
“Soto, I. and Lagos, C.”, Title: “System and method for communication by means of visible light for underground tunnels”. Country: USA, *N*°: US2019/0007143A1.
“Soto, I. and Lagos, C.”, Title: “Sistema y método de comunicación a través de luz visible para túneles subterráneos”. Country: COLOMBIA, *N*°: Colombian Patent 36068.

## Abbreviations

The following abbreviations are used in this manuscript:

ADC	Analogic-Digital Converter
COO	Cell of Origin
CPE	Convex Position Estimation
DD	Direct Detection
DR	Dead Reckoning
EPS	Electric Power System
FOV	Field of View
GNSS	Global Navigation Satellite System
GPS	Global Positioning System
IFSC	Federal Institute of Santa Catarina
IM	Intensity Modulation
IMU	Inertial Measurement Unit
INS	Inertial Navigation Systems
IR	Infrared
LED	Light-Emitting Diode
Li-Fi	Light Fidelity
LOS	Line of Sight
MF	Medium Frequency
OWC	Optical Wireless Communication
PD	Photodetector
PDC	Phasor Data Concentrator
PMU	Phasor Measurement Unit
RF	Radio Frequency
Rx	Receiver
SCADA	Supervisory Control and Data Acquisition
SHF	Super High Frequency
SPMS	Synchronized Phasor Measurement System
Tx	Transmitter
UFSC	University of Santa Catarina
UHF	Ultra High Frequency
USACH	University of Santiago of Chile
UTC	Coordinated Universal Time
VHF	Very High Frequency
VLC	Visible Light Communication
VLP	Visible Light Positioning
VSAT	Very Small Aperture Trminal
Wi-Fi	Wireless Fidelity
